# Distributed Power-Line Outage Detection Based on Wide Area Measurement System

**DOI:** 10.3390/s140713114

**Published:** 2014-07-21

**Authors:** Liang Zhao, Wen-Zhan Song

**Affiliations:** Department of Computer Science, Georgia State University, 34 Peachtree Street, Atlanta, GA 30329, USA; E-Mail: wsong@gsu.edu

**Keywords:** line outage detection, convex optimization, smart grid, distributed computing, alternating direction method of multipliers

## Abstract

In modern power grids, the fast and reliable detection of power-line outages is an important functionality, which prevents cascading failures and facilitates an accurate state estimation to monitor the real-time conditions of the grids. However, most of the existing approaches for outage detection suffer from two drawbacks, namely: (i) high computational complexity; and (ii) relying on a centralized means of implementation. The high computational complexity limits the practical usage of outage detection only for the case of single-line or double-line outages. Meanwhile, the centralized means of implementation raises security and privacy issues. Considering these drawbacks, the present paper proposes a distributed framework, which carries out in-network information processing and only shares estimates on boundaries with the neighboring control areas. This novel framework relies on a convex-relaxed formulation of the line outage detection problem and leverages the alternating direction method of multipliers (ADMM) for its distributed solution. The proposed framework invokes a low computational complexity, requiring only linear and simple matrix-vector operations. We also extend this framework to incorporate the sparse property of the measurement matrix and employ the LSQRalgorithm to enable a warm start, which further accelerates the algorithm. Analysis and simulation tests validate the correctness and effectiveness of the proposed approaches.

## 1. Introduction

The evolving modern “smart grid” is devoted to leveraging information and communication technologies to enrich the efficiency, reliability and sustainability of the operation of the energy distribution. Particularly, the advances in information infrastructure provide opportunities to better cope with the reliability issues. For instance, phasor measurement units (PMUs) are deployed to get the complex voltages and currents directly, and smart meters are implemented between end-users and the distribution networks for the collection and processing of information [1]. These ample kinds of sensors offer much more powerful potential monitoring capabilities than a traditional grid. However, efficient and effective ways of data communication, computation and inference become key challenges to the success of a smart grid.

On the other hand, the smart grid has been regarded as an integration of computation, networking and control for a physical power grid in which the physical system can affect the cyber system and *vice versa*. It is said that a smart grid forms a rich environment for the study of several inherent problems. In the first place, it becomes one of the largest and most complex interconnected networks in the world and the corresponding control tasks are extremely difficult, due to its vast scale. Second, new kinds of power transfers, resulting from the use of distributed energy generation and storage, will potentially make power systems increasingly vulnerable to cascading failures, in which a series of small vibrations could lead to a major blackout [2]. Thus, an advanced smart grid system calls for a framework integrating distributed computation, communication and control, in which local actions can be coordinated for the effective protection of the power grid as a whole.

A key aspect of situational awareness in the power grid is the knowledge of transmission line status. Lessons learned from the 2003 northeastern blackout in United States reveal that accurate line monitoring in real-time is required throughout the whole power grid [3]. Fortunately, the development of real-time synchronized PMUs enables the direct usage of PMU-provided measurements to detect events within the power grid. At present, PMU-based line outage detection has been considered as a promising approach to facilitate effective fault identification.

In this paper, we aim at proposing a scheme to detect power-line outage in a distributed manner. The proposed scheme relies on a wide area measurement system (WAMS), which can be seen as a network of sensors that cooperatively measure the status of the grid. The proposed scheme is expected to work based on WAMS as follows. First, the raw measurements from different PMUs are collected in the corresponding phasor data concentrators (PDCs) for processing; second, the line outage detection is performed among the PDCs in a distributed fashion; finally, the results after detection (instead of the raw data) are transmitted to the WAMS center, which provides critical information to the system operators.

The rest of the paper is organized as follows. Section 2 presents the the related work of line outage detection in a smart grid and distributed diagnosis in other applications. In Section 3, we summarize the specifications and assumptions in the proposed framework. The problem formulation and related preliminaries are discussed in Section 4. The design of distributed algorithms is presented in Section 5. In Section 6, we analyze and discuss the simulation results. We then conclude the paper in Section 7.

## 2. Related Work

Existing PMU-based line outage detection methods typically use the internal-external network model for the whole interconnected system in which the goal is to identify external line outages using only measurements within the internal system [4–8]. Specifically, [6] formulates line outage detection as a best match problem, which contains an exhaustive searching process for the most likely outage line. Thus, it can only handle the single-line outage scenario. Building upon the work of [6], double-line outage detection is considered in [7], while it restricts to the case with exactly a double-line outage in the system. A similar exhaustive search is also applied in [7], but the searching space is much larger than that of the single-line case, which, thus, is very computationally expensive. Another method for line outage identification employs a Gauss–Markov graphical model of the power network and is capable of dealing with multiple outages at a moderate complexity [9] despite requiring a grid-wise measurement. An alternative sparse overcomplete representation-based algorithm was proposed in [8], which can also handle multiple line outages. Then, Chen *et al.* developed a global stochastic optimization technique based on cross-entropy optimization [10]. The algorithm in [10] does not require prior knowledge of the parameters used in [8], whose selection can significantly affect solution accuracy. Wu *et al.* proposed an ambiguity group-based location recognition algorithm, which claims to be faster and shows higher accuracy than the algorithm in [8] for multiple line outage detection [11]. Banerjee *et al.* exploited the fact that the line outage is persistent and studied the problem of line outage detection and identification in the framework of the theory of quickest change detection [12]. However, the aforementioned methods all carry out the processing in a centralized manner, which is vulnerable in practice. Further, these existing approaches need to transmit raw data in the system and, thus, may raise privacy issues.

Huge recent interest in research and applications fall into distributed methods for diagnosing faults in complex distributed systems. In [13], a distributed fault detection method was devised for rail vehicle suspension systems in which the observers are co-operated mainly by the state estimation errors. A hidden Markov random field-based distributed fault detection algorithm was invented for wireless sensor networks [14].

Our key contributions in this paper can be summarized as follows:

- We formulate the line outage detection problem in a smart grid as a convex optimization problem, which can be solved efficiently in practice.
- We propose a distributed algorithm to solve the aforementioned problem by using the alternative direction multiplier method (ADMM). It overcomes the computational burden and privacy issues. This approach requires only simple matrix-vector operations, which is compatible with real power grids.
- An improved LSQR-based warm-started, distributed line change detection is developed, which can speed up the previous ADMM-based distributed algorithm.

## 3. Specifications for the Proposed Framework

Our main idea is to devise a distributed and robust protocol that can be performed in WAMS for smart grid monitoring application. In this section, the assumptions and problem settings in the proposed method will be described.

### 3.1. Sensor Network Model

**Figure 1.**
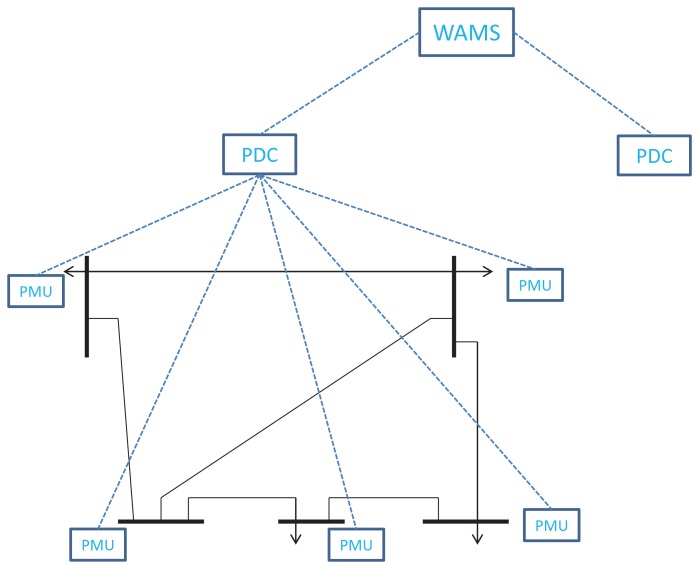
Hierarchical architecture of a wide area measurement system (WAMS) in a smart grid.

Our proposed method is based on the hierarchical network of WAMS (as shown in Figure 1), which consists of a hierarchical structure, as follows. In each area, a certain number of PMUs are installed in the bus substations of the power grid. In the middle level, there is a set of phasor data concentrators (PDCs). Each PDC can share information with the PDCs in neighborhoods. In the top level, there is a WAMS center, which collects information from PDCs supporting the system-wide monitoring task. As a result, we can naturally see that in each area with a PDC, it is a local control area or sub-system [15].

### 3.2. Sensor Measurement Settings

We consider a linear physical equation describing the relation between the measurable quantity and the set of unknown variables. The set of unknown variables is related to the sensor reading through the measurement matrix. In this paper, the branch currents are considered as the unknown quantities, and the measurements we use are bus voltage phasors and all the branch-current phasors that are incident to the bus if a PMU is installed in the bus substation. Our algorithm recognizes faulty/normal lines by determining whether their linear physical measurement equations are valid or not. Furthermore, an additional assumptions is made:

- For our purpose of detecting possible faulty lines, the number of measurements we have is relatively smaller than the number of unknown variables, which implies that the measurement matrix is under-determined.

## 4. Problem Formulation

In this section, we describe the detailed measurement equation and centralized line outage detection solution adopted in this paper. The proposed novel algorithm will be built upon them.

### 4.1. PMU Measurement Equation

In a typical power transmission system, the synchrophasor measurements at the **n**-th PDC area, expressed in rectangular coordinates, are collected in a vector **ȳ_n_**, and they satisfy the following linear model:

(1) $${\bar{\text{y}}}_{\text{n}}={\bar{\text{H}}}_{\text{n}}\text{x}+{\bar{\text{g}}}_{\text{n}}$$

where **x** is the unknown vector to be estimated containing all branch currents, **H̄_n_** ∈ ℝ^*M_n_*×2*N_l_*^ is the measurement matrix, *M_n_* is the number of measurements within the **n**-th PDC area, *N_l_* is the number of transmission lines in the whole system and **ḡ_n_** ∼ 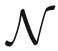
(**0**, **Λ_n_**) denotes the additive Gaussian noise vector. For notational convenience, we multiply with $\Lambda_{\text{n}}^{-1/2}$ on both sides of Equation (1) to yield:

(2) $${\text{y}}_{\text{n}}={\text{H}}_{\text{n}}\text{x}+{\text{g}}_{\text{n}}$$

where ${\text{y}}_{\text{n}}=\Lambda_{\text{n}}^{-1/2}{\bar{\text{y}}}_{\text{n}}$, and the other terms are manipulated similarly. Using Equation (2), the weighted least squares form:

$${\left\|\Lambda_{\text{n}}^{-1/2}({\bar{\text{y}}}_{\text{n}}-{\bar{\text{H}}}_{\text{n}}\text{x})\right\|}_{2}^{2}$$

is replaced by the regular least squares ${\left\|{\text{y}}_{\text{n}}-{\text{H}}_{\text{n}}\text{x}\right\|}_{2}^{2}$. We will use this notation in the following sections.

Now, we first introduce some basic concepts on electrical circuits:

- Kirchhoff's current law: at any node (junction) in an electrical circuit, the sum of currents flowing into that node is equal to the sum of currents flowing out of that node.
- Kirchhoff's voltage law: the sum of all voltage drops and rises in a closed loop equals zero.

The laws above are two approximate equalities that deal with the current and voltage difference in electrical circuits [16].

Let **v** = *Re*(**v**) + *Im*(**v**) be the *N_b_* × 1 vector of complex nodal voltages with *N_b_* the number of buses in the system. By writing down the node equations of Kirchhoff's current law (KCL) and Kirchhoff's voltage law (KVL) at each node, we can derive the vector of complex currents injected on each line as follows:

(3) $${\text{i}}_{\text{fl}}\;=\;\text{x}\;=\;{\text{Y}}_{\text{fl}}\;\text{v}$$

where **y_fl_** describes the line-to-bus admittance matrix. The matrices **H̄_n_** in Equation (1) can be expressed as:

(4) $${\bar{\text{H}}}_{\text{n}}=\left(\begin{array}{cc} {\text{Q}}_{\text{n}}\text{Re}{(\text{Y}}_{\text{fl}}^{-1}\text{)} & -{\text{Q}}_{\text{n}}\text{Im}{(\text{Y}}_{\text{fl}}^{-1}\text{)}\\ {\text{Q}}_{\text{n}}\text{Im}{(\text{Y}}_{\text{fl}}^{-1}\text{)} & {\text{Q}}_{\text{n}}\text{Re}{(\text{Y}}_{\text{fl}}^{-1}\text{)}\\ {\text{e}}_{\text{n}}^{\text{T}} & 0^{\text{T}}\\ 0^{\text{T}} & {\text{e}}_{\text{n}}^{\text{T}} \end{array}\right)$$

where **Q_n_** is the selection matrix according to the **n**-th PDC.

At this point, our problem is equivalent to using a distributed method to determine whether the linear model in Equation (1) is valid. A conventional and straightforward way to solve this problem would be:

(1) In each PDC area, estimate the unknown variables locally.
(2) Communicate and share the estimates with other PDCs.
(3) Perform a fusion of estimates in each PDC.
(4) Apply a likelihood ratio test to detect faulty lines.

This above method will work well when there are sufficient measurements (more than the number of unknown variables) available in each PDC [17]. However, in some scenarios, for example in the smart grid system that we focus on in this paper, fetching sufficiently-sized measurements may be infeasible or costly. Consequently, a framework that can make accurate decisions with fewer data sets will be of practical importance. From the next section, we are going to describe our solution for this purpose.

### 4.2. Possible Centralized Solution for Line Outage Detection

In this paper, we combine the measurements and the prior information on the branch currents to do the line outage detection. We consider a Bayesian framework, where the branch current variables are random vectors with Gaussian distribution 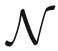
(**x_p_**, **Λ_p_**). We assume that, in practice, the mean vector **x_p_** and covariance matrix **Λ_p_** can be estimated from historical data [18,19]. The variables are assumed to be independent, and thus, the covariance matrix **Λ_p_** is diagonal. Inspired by the idea of compressive sensing, we can have a sparse solution for a certain under-determined system by adding the ℓ_1_-norm regularization [20]. Since most of the components of the item in the ℓ_1_-norm term is pushed into zero, we make the unknown vector x to compare with its nominal model in the ℓ_1_-norm term in order to create “sparse” faulty branches. Now, suppose that there are k transmission line outages in the system. Then, the maximum likelihood (ML) estimation in a single control center can be formulated as:

(5) $$\begin{array}{ll} \underset{\text{x}}{\text{minimize}} & \frac{1}{2}{\left\|\text{y}-\text{Hx}\right\|}_{2}^{2}\\ \text{subject}\;\text{to}: & {\left\|\Lambda_{\text{p}}^{-1/2}(\text{x}-{\text{x}}_{\text{p}})\right\|}_{0}=k \end{array}$$

where **x** is the unknown vector of the system defined in Equation (1). **y** denotes the measurements collected in the single center, and **H** is the corresponding measurement matrix of the system. It contains the global topology and impedance information. Note that ‖· ‖*_p_* means *p*-norm. Here, the faulty lines can be identified by non-zero components in the vector **x** – **x_p_**. Based on the optimization theory [21], there exists a λ that makes the following equation equivalent to the problem formulation (5):

(6) $$\underset{\text{x}}{\min}\frac{1}{2}{\left\|\text{y}-\text{Hx}\right\|}_{2}^{2}+\lambda {\left\|\Lambda_{\text{p}}^{-1/2}(\text{x}-{\text{x}}_{\text{p}})\right\|}_{0}$$

where λ > 0 is an application-dependent pre-defined parameter. It quantizes the tradeoff of effects between the two objectives in Equation (6). The selection of λ will be discussed in a later section.

Both Equations (5) and (6) are non-convex, which means it is hard to solve them exactly in a reasonable time. We employ the ℓ_1_-norm approximation in [20] to replace the zero-norm term in Equation (6), which leads to the convex optimization problem shown below:

(7) $$\underset{\text{x}}{\min}\frac{1}{2}{\left\|\text{y}-\text{Hx}\right\|}_{2}^{2}+\lambda {\left\|\Lambda_{\text{p}}^{-1/2}(\text{x}-{\text{x}}_{\text{p}})\right\|}_{1}$$

#### Remark 1

*The centralized grid-wise measurement data collection the computation in implementing Equation* (7) *are inefficient due to bandwidth and time constraints or infeasible because of data privacy concerns; thus, distributed computations are strongly preferred or demanded*.

## 5. Distributed Line Outage Detection

In this section, we striveto solve the optimization problem in Equation (7) in a distributed manner. Note that if we decompose Equation (7) into N PDC areas, then Equation (7) can be expressed in the following:

(8) $$\underset{{\text{x}}_{\text{n}}}{\min}\overset{N}{\underset{n=\text{1}}{\text{∑}}}f_{\text{n}}({\text{x}}_{\text{n}})$$

in which function *f***_n_**(**x_n_**) denotes the “cost function” for each PDC, and it is given by:

(9) $$f_{\text{n}}({\text{x}}_{\text{n}})=\frac{1}{2}{\left\|{\text{y}}_{\text{n}}-{\text{H}}_{\text{n}}{\text{x}}_{\text{n}}\right\|}_{2}^{2}+\lambda {\left\|\Lambda_{\text{pn}}^{-1/2}({\text{x}}_{\text{n}}-{\text{x}}_{\text{pn}})\right\|}_{1}$$

where **x_n_**, **H_n_**, **x_pn_** and **Λ_pn_** correspond to the unknown variables associated with the **n**-th PDC. Each PDC in the area can choose to minimize Equation (9) individually, but this method is clearly sub-optimal, since the overlapping variables are not taken into account.

### Remark 2

*The criterion in Equation* (9) *will force some entries of the vector of branch currents (***x_n_***) equal to their mean values (corresponding entries of* **x_pn_***)*, *which implies that they are consistent with their statistical distribution, and thus, these branches are recognized as in the normal condition. On the other hand, if certain entries of the branch currents fail to be equal to their mean values, then the associated branches are considered to be possibly faulty or abnormal*.

### 5.1. Distributed Power-Line Change Detection Solution

**Figure 2.**
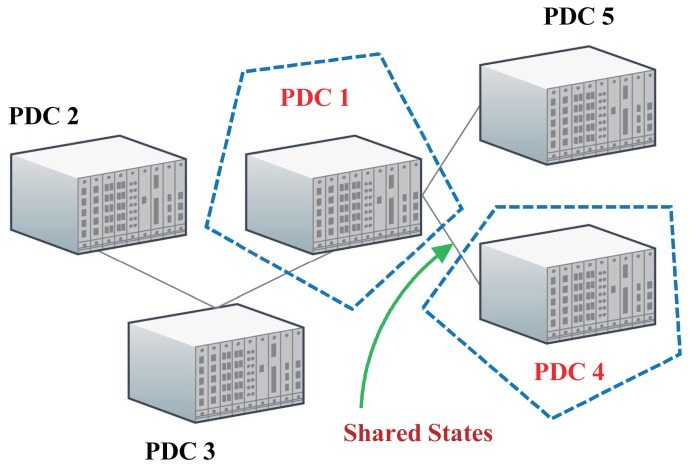
An example of a phasor data concentrator (PDC) network.

Denote **x_n_** as the sub-vector of **x**, which contains the unknown variables involved in the **n**-th PDC. Furthermore, denote **x_nm_** as the value of the shared variables between neighboring **n**-th and **m**-th PDC (a sub-vector of **x_n_** or **x_m_**). Then, the estimate of overlapping unknown variables by neighboring PDCs should be same. Then, Equation (8) can be reformulated as:

(10) $$\begin{array}{ll} \underset{{\text{x}}_{\text{n}}}{\text{minimize}} & \overset{N}{\underset{\text{n}=1}{\text{∑}}}f_{\text{n}}({\text{x}}_{\text{n}})\\ \text{subject}\;\text{to}: & {\text{x}}_{\text{nm}}={\text{x}}_{\text{mn}}=\text{m}\in N_{\text{n}};\text{n},\text{m}\in P \end{array}$$

where 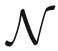
_n_ is the set of neighboring PDCs of the **n**-th PDC and *P* is the set of PDCs. For instance, in Figure 2, Node 1 and Node 4 share the edge (1,4). This means that these two PDC areas have overlapping unknown variables. As a result, Node 1's estimate of the branch current on (1,4) should be the same as Node 4's estimate on (1,4).

In this paper, the proposed formulation for line outage detection in Equation (10) is solved by resorting to the so-called ADMM. To briefly illustrate the general ADMM algorithm [22], consider the prototype problem:

(11) $$\begin{array}{ll} \text{minimize} & f(x)+g(z)\\ \text{subject}\;\text{to}: & Ax+Bz=c \end{array}$$

with variables *x* ∈ **R***^n^* and *z* ∈ **R***^m^*, where *A* ∈ **R***^p^*^×^*^n^*, *B* ∈ **R***^p^*^×^*^m^* and *c* ∈ **R***^p^*. Functions *f* and *g* are assumed to be convex. As in the method of multipliers, the augmented Lagrangian can be formed:

$$L_{\rho }(x,z,y)=f(x)+g(z)+y^{T}(Ax+Bz-c)+(\rho /2){\left\|Ax+Bz-c\right\|}_{2}^{2}$$

ADMM consists of the iterations:

(12a) $$x^{k+1}:=\underset{x}{\arg\min}L_{\rho }(x,z^{k},y^{k})$$

(12b) $$z^{k+1}:=\underset{z}{\arg\min}L_{\rho }(x^{k+1},z,y)$$

(12c) $${\text{y}}^{k+1}:=y^{k}+\rho (Ax^{k+1}+Bz^{k+1}-c)$$

where *ρ* > 0 is the predefined augmented Lagrangian parameter and *y* is the Lagrangian multiplier (dual variable) of the constraint in Equation (11). The ADMM algorithm is considered to have three steps: an *x*-minimization Equation (12a), a *z*-minimization step Equation (12b) and a dual variable update Equation (12c).

Let us now apply the method of ADMM in [22] to solve the line outage detection problem formulated in Equation (10) using a distributed mechanism. We introduce auxiliary variables *ϑ***_nm_** and **z_n_** in order to fit the ADMM framework. Then, Equation (10) can be alternatively expressed as:

(13) $$\begin{array}{ll} \underset{{\text{x}}_{\text{n}},\vartheta_{\text{nm}},{\text{z}}_{\text{n}}}{\text{minimize}} & \overset{N}{\underset{\text{n}=1}{\text{∑}}}f_{\text{n}}({\text{x}}_{\text{n}})\\ \text{subject}\;\text{to}: & {\text{x}}_{\text{nm}}=\vartheta_{\text{nm}},\text{m}\in N_{\text{n}};\text{n},\text{m}\in P\\ & {\text{x}}_{\text{n}}-{\text{x}}_{\text{pn}}={\text{z}}_{\text{n}} \end{array}$$

We also introduce variable *ν***_nm_** to denote the Lagrangian multiplier for the first constraint in Equation (13) and **s_n_** to denote the multiplier for the second constraint in Equation (13). Note that by using ADMM in our problem, there are three primal variables: **x_n_**, *ϑ***_nm_** and **z_n_**; two dual variable: *ν***_nm_** and **s_n_**. The augmented Lagrangian function can be obtained as:

(14) $$\begin{array}{l} L_{\rho }({\text{x}}_{\text{n}},\vartheta_{\text{nm}},{\text{z}}_{\text{n}},v_{\text{nm}},{\text{s}}_{\text{n}})\\ =\overset{\text{N}}{\underset{\text{n}=1}{\text{∑}}}\{f_{\text{n}}({\text{x}}_{\text{n}})+\underset{\text{m}\in N_{\text{n}}}{\text{∑}}\left(v_{\text{nm}}^{\text{T}}({\text{x}}_{\text{nm}}-\vartheta_{\text{nm}}\right)\\ +(\rho /2){\left\|{\text{x}}_{\text{nm}}-\vartheta_{\text{nm}}\right\|}_{2}^{2})+{\text{s}}_{\text{n}}^{T}({\text{x}}_{\text{n}}-{\text{x}}_{\text{pn}}-{\text{z}}_{\text{n}})\\ +(\rho /2){\left\|{\text{x}}_{\text{n}}-{\text{x}}_{\text{pn}}-{\text{z}}_{\text{n}}\right\|}_{2}^{2}\} \end{array}$$

Let *k* be the iteration index; then, the ADMM algorithm consists of the following update rules:

(15a) $${\text{x}}_{\text{n}}^{k+1}=\underset{{\text{x}}_{\text{n}}}{\arg\min}L_{\rho }({\text{x}}_{\text{n}},\vartheta_{\text{nm}}^{k},{\text{z}}_{\text{n}}^{k},v_{\text{nm}}^{k},{\text{s}}_{\text{n}}^{k})$$

(15b) $$\left(\vartheta_{\text{nm}}^{k+1},{\text{z}}_{\text{n}}^{k+1})=\underset{\vartheta_{\text{nm}}{,\text{z}}_{\text{n}}}{\arg\min}L_{\rho }({\text{x}}_{\text{n}}^{k+1},\vartheta_{\text{nm}},{\text{z}}_{\text{n}},v_{\text{nm}}^{k},{\text{s}}_{\text{n}}^{k}\right)$$

(15c) $$v_{\text{nm}}^{k+1}=v_{\text{nm}}^{k}+\rho ({\text{x}}_{\text{nm}}^{k+1}-\vartheta_{\text{nm}}^{k+1})\text{for}\;\text{all}\;\text{n},\;\text{m}\text{.}$$

(15d) $${\text{s}}_{\text{n}}^{k+1}={\text{s}}_{\text{n}}^{k}+\rho ({\text{x}}_{\text{n}}^{k+1}-{\text{x}}_{\text{pn}}-{\text{z}}_{\text{n}}^{k+1})$$

To simplify the presentation, we combine the linear and quadratic terms in the augmented Lagrangian in Equation (14) that can be applied in Equations (15a) and (15b) by ignoring the terms independent of the decision variables:

(16) $$\begin{array}{l} L_{\rho }({\text{x}}_{\text{n}},\vartheta_{\text{nm}},{\text{z}}_{\text{n}},v_{\text{nm}}^{k},{\text{s}}_{\text{n}}^{k})=\\ \overset{N}{\underset{\text{n}=1}{\text{∑}}}(f_{\text{n}}({\text{x}}_{\text{n}})+\underset{\text{m}\in N_{\text{n}}}{\text{∑}}(\rho /2){\left\|{\text{x}}_{\text{nm}}-\vartheta_{\text{nm}}+(1/\rho )v_{\text{nm}}^{k}\right\|}_{2}^{2}\\ +(\rho /2){\left\|{\text{x}}_{\text{n}}-{\text{x}}_{\text{pn}}-{\text{z}}_{\text{n}}+(1/\rho ){\text{s}}_{\text{n}}^{k}\right\|}_{2}^{2}) \end{array}$$

Now, we are concerned about how to implement the updates in Equations (15a)–(15d) efficiently. Since Equations (15c) and (15d) are simple linear updating equations, we only need to focus on the deduction of Equations (15a) and (15b). To solve Equation (15a), several algebraic manipulations are used to enable the simplification of the analysis. We define:

(1) **D_n_** as a diagonal matrix with its (**m**,**m**)-th entry being 1;
(2) ${\text{r}}_{\text{n}}^{k}=\vartheta_{\text{n}}^{k}-(1/\rho )v_{\text{n}}^{k}$
(3) **I_n_** denotes an identity matrix with its dimension being the number of states in **n**-th area.

As a result, the term $\underset{\text{m}\in N_{\text{n}}}{\text{∑}}\left(\frac{\rho }{2}\right){\left\|{\text{x}}_{\text{nm}}-\vartheta_{\text{nm}}+\left(\frac{1}{\rho }\right)v_{\text{nm}}^{k}\right\|}_{2}^{2}$ in Equation (15a) can be expressed as: $(\rho /2){\left\|{\text{D}}_{\text{n}}({\text{x}}_{\text{n}}-{\text{r}}_{\text{n}}^{k})\right\|}_{2}^{2}$. Then, after manipulating via matrix calculus, we obtain the minimizer of Equation (15a) as follows:

(17) $$\begin{array}{l} {\text{x}}_{\text{n}}^{k+1}={\left({\text{H}}_{\text{n}}^{T}{\text{H}}_{\text{n}}+\rho {\text{D}}_{\text{n}}+\rho {\text{I}}_{\text{n}}\right)}^{-1}\\ \times ({\text{H}}_{\text{n}}^{T}{\text{y}}_{\text{n}}+\rho ({\text{D}}_{\text{n}}{\text{r}}_{\text{n}}^{k}+{\text{x}}_{\text{pn}}+{\text{z}}_{\text{n}}^{k}-(1/\rho ){\text{s}}_{\text{n}}^{k})) \end{array}$$

Regarding solving Equation (15b), it is known that the optimality conditions satisfy when the zero vector belongs to subdifferentials of Equation (15b) with respect to variable *ϑ***_nm_** and **z_n_** [23]. We first consider the minimization with *ϑ***_nm_**; the following Theorem is derived in order to conclude the updates of *ϑ***_nm_**.

#### Theorem 1

*For each pair of* **n**, **m** *in Equation* (15c), *the following holds for the updating Lagrange multipliers:* $v_{\text{nm}}^{k}+v_{\text{mn}}^{k}=0$

#### Proof

In Equation (15b), we note that the optimization task will be performed in **n**-th and **m**-th PDC in parallel for each adjacent pair (**n**, **m**). Thus, we can obtain the following result by solving Equation (15b) for (**n**, **m**) and (**m**, **n**), respectively:

(18) $$\begin{array}{l} \vartheta_{\text{nm}}^{k+1}={\text{x}}_{\text{nm}}^{k+1}+(1/\rho )v_{\text{nm}}^{k}\\ \vartheta_{\text{mn}}^{k+1}={\text{x}}_{\text{mn}}^{k+1}+(1/\rho )v_{\text{mn}}^{k} \end{array}$$

where *ϑ***_nm_** and *ϑ***_mn_** are the same variable; then, averaging the both sides of the two equations in Equation (18) implies:

(19) $$\vartheta_{\text{nm}}^{k+1}=(\frac{{\text{x}}_{\text{nm}}^{k+1}+{\text{x}}_{\text{mn}}^{k+1}}{2})+(\frac{v_{\text{nm}}^{k}+v_{\text{mn}}^{k}}{2\rho })$$

In a similar manner, we can express $\vartheta_{\text{nm}}^{k+1}$ and $\vartheta_{\text{mn}}^{k+1}$ by using Equation (15c). The calculations are:

(20) $$\begin{array}{l} \vartheta_{\text{nm}}^{k+1}={\text{x}}_{\text{nm}}^{k+1}+(1/\rho )v_{\text{nm}}^{k}-(1/\rho )v_{\text{nm}}^{k+1}\\ \vartheta_{\text{mn}}^{k+1}={\text{x}}_{\text{mn}}^{k+1}+(1/\rho )v_{\text{mn}}^{k}-(1/\rho )v_{\text{mn}}^{k+1} \end{array}$$

Finally, averaging both sides of Equation (20) yields:

(21) $$\begin{array}{ll} \vartheta_{\text{nm}}^{k+1} & =(\frac{\vartheta_{\text{nm}}^{k+1}+\vartheta_{\text{mn}}^{k+1}}{2})\\ & =(\frac{{\text{x}}_{\text{nm}}^{k+1}+{\text{x}}_{\text{mn}}^{k+1}}{2})+(\frac{v_{\text{nm}}^{k}+v_{\text{mn}}^{k}}{2\rho })\\ & -(\frac{v_{\text{nm}}^{k+1}+v_{\text{mn}}^{k+1}}{2\rho }) \end{array}$$

By comparing the right side of Equations (19) and (21), we find that the only different part is the last item in Equation (21), which turns out to be zero. Theorem 1 is then proven.

At this point, it is clear to see that by using Theorem 1, Equation (19) can be reduced to:

(22) $$\vartheta_{\text{nm}}^{k+1}=\frac{\left({\text{x}}_{\text{nm}}^{k+1}+{\text{x}}_{\text{mn}}^{k+1}\right)}{2}$$

Next, we are concerned about how to address the updates of **z_n_**. Note that due to the ℓ_1_-norm term, Equation (15b) is not differentiable everywhere, but sub-differentiable with respect to **z_n_** [23]. As mentioned previously, we take the sub-differential over Equation (15b) with respect to **z_n_** and the optimality condition becomes:

$$0\in \partial \lambda {\left\|\Lambda_{\text{pn}}^{-1/2}{\text{z}}_{\text{n}}\right\|}_{1}+\rho ({\text{z}}_{\text{n}}-({\text{x}}_{\text{n}}^{k+1}-{\text{x}}_{\text{pn}}+(1/\rho ){\text{s}}_{\text{n}}^{k}))$$

By using the soft thresholding operator defined in [22], for instance, the *i*-th component ${\text{z}}_{\text{n}}^{k+1}[i]$ (scalar) is updated as:

$${\text{z}}_{\text{n}}^{k+1}[i]=S_{\left(\lambda /\rho )\Lambda_{\text{pn}}^{-1/2}[i][i\right]}({\text{x}}_{\text{n}}^{k+1}[i]-{\text{x}}_{\text{pn}}[i]+(1/\rho ){\text{s}}_{\text{n}}^{k}[i])$$

In a similar way, a closed-form solution for the updates of **z_n_** is obtained as follows:

(23) $${\text{z}}_{\text{n}}^{k+1}[i]=S_{(\lambda /\rho )\Lambda_{\text{pn}}^{-1/2}}({\text{x}}_{\text{n}}^{k+1}-{\text{x}}_{\text{pn}}+(1/\rho ){\text{s}}_{\text{n}}^{k})$$

where:

(24) $$S_{b}(a)=\{\begin{array}{ll} a-b, & a>b;\\ 0, & \left|a\right|\leq b;\\ a+b, & a<-b. \end{array}$$

Note, here, component-wise updating is applied, such that the *i*-th component of **z_n_** is updated according to the *i*-th entry of the rest of the vectors in Equation (23) and the (*i*, *i*)-th entry of the diagonal matrix $\Lambda_{\text{pn}}^{-1/2}$.

Now, the ADMM updating in Equations (15a)–(15d) for each processor can be summarized in Algorithm 1.


**Algorithm 1 Distributed line change detection (D-LCD).**
1:**Input: y_n_**, **H_n_**, **Λ_n_**, **Λ_pn_**, **x_pn_**, **D_n_**, λ > 0, *ρ* > 0, *k* = 0.2:Initialize: **x_n_**, *ϑ***_nm_**, **z_n_**, *ν***_nm_**, **s_n_**.3:**while** not converged or stopping criterion not reached **do**4:*k* ← *k* + 1.5:Update 
${\text{x}}_{\text{n}}^{k+1}$ based on Equation (17).6:Exchange 
${\text{x}}_{\text{nm}}^{k+1}$ with its neighbors.7:Update 
$\vartheta_{\text{nm}}^{k+1}$, 
$z_{\text{n}}^{k+1}$ via Equations (22) and (23), respectively.8:Update 
${\text{v}}_{\text{nm}}^{k+1}$ and 
${\text{s}}_{\text{n}}^{k+1}$ through Equations (15c) and (15d).9:**end while**


### 5.2. Distributed Line Change Detection with Warm Start

The most computational-intensive step in Algorithm 1 is the update of **x_n_** given in Equation (17), which, in essence, requires matrix inversion and multiplication for each PDC in every iteration. Nevertheless, a detailed look shows that the variables in Equation (17) may not change significantly within two consecutive iterations. The previous ADMM iteration ${\text{x}}_{\text{n}}^{k}$ often provides a good approximation to the results, which can be used as a warm start to update ${\text{x}}_{\text{n}}^{k}$. The warm start process reduces the complexity in computing ${\text{x}}_{\text{n}}^{k+1}$, since the computation starts from a more appropriate initialization instead of from zero (or some other fixed and default initialization) [22].

Now, if we look at the the minimization step in Equation (15a) along with its minimizer in Equation (17), it actually can be regarded as solving a system of linear equations:

(25) Ax=b

The least squares solution of Equation (25) is [24]:

(26) $$\text{x}={\left({\text{A}}^{T}\text{A}\right)}^{-1}{\text{A}}^{T}b$$

We observe that Equation (17) is equivalent to finding the least squares solution with matrix **A** and vector **b** formed in the following:

(27) $$\text{A}=\left(\begin{array}{c} {\text{H}}_{\text{n}}\\ \sqrt{\rho }{\text{I}}_{\text{n}}\\ \sqrt{\rho }{\text{D}}_{\text{n}} \end{array}\right)$$

(28) $$\text{b}=\left(\begin{array}{c} {\text{y}}_{\text{n}}\\ \sqrt{\rho }{(\text{x}}_{\text{pn}}{+\text{z}}_{\text{n}}^{k}-\frac{1}{\rho }{\text{s}}_{\text{n}}^{k}\text{)}\\ \sqrt{\rho }{\text{r}}_{\text{n}}^{k} \end{array}\right)$$

**Algorithm 2 D-LCD with a warm start.**
1:**Input: y_n_**, **H_n_**, **Λ_n_**, **Λ_pn_**, **x_pn_**, **D_n_**, λ > 0, *ρ* > 0, *k* = 0.2:Initialize: **x_n_**, *ϑ***_nm_**, **z_n_**, *ν***_nm_**, **s_n_**.3:**while** not converged or stopping criterion not reached **do**4:Assemble **A** and **b** according to Equations (27) and (28).5:Solve linear Equations **Ax** = **b** using LSQR procedure with initial value 
${\text{x}}_{\text{n}}^{k}$.6:*k ← k* + 1.7:Update 
${\text{x}}_{\text{n}}^{k+1}$ based on the solution in Step 5.8:Exchange 
${\text{x}}_{\text{nm}}^{k+1}$ with its neighbors.9:Update 
$\vartheta_{\text{nm}}^{k+1}$, 
$z_{\text{n}}^{k+1}$ via Equations (22) and (23), respectively.10:Update 
${\text{v}}_{\text{nm}}^{k+1}$ and 
${\text{s}}_{\text{n}}^{k+1}$ through Equations (15c) and (15d).11:**end while**


At this point, we have changed the problem of **x_n_**-update in Equation (17) into finding a method to solve linear equations in Equation (25) with **A** and **b** defined in Equations (27) and (28), respectively. To this end, we adopt the LSQR algorithm in this paper. Recall that **I_n_**, **D_n_** are diagonal matrices and that **H_n_** is sparse in general. Thus, matrix **A** is also sparse. LSQR thus fits our need, since it is very efficient for solving sparse linear equations [25]. Interested readers, please refer to [25] for the details. We omit its introduction, here due to space limitation. In short, the modified distributed line detection algorithm with a warm start is described in Algorithm 2.

### 5.3. Selection of the Tuning Parameter

In our proposed centralized and distributed algorithms stated in Equation (7) and Algorithm 1, we have to choose the parameter λ first. As discussed in Section 4.2, ℓ_1_-norm term in Equation (7) will force the item in the norm to be sparse, and λ determines the importance of this objective. If λ is very large, most of the components in the ℓ_1_-norm would be zeros. In other words, the tuning parameter λ specifies the sparsity level of the solution. In addition, the selection of λ depends on the specific application we are working on. Thanks to the help of the cross-validation technique, we can have some portion of data for model validation. The optimized λ is then derived in terms of prediction accuracy. By using the “one-standard-error” rule, one can also have the largest value of λ, such that the error is within one standard-error of the minimum [26].

## 6. Numerical Tests

To evaluate the proposed centralized and distributed line change detection algorithms, we use an Intel Duo Core at 1.8 GHz (1.5 GB RAM) computer with MATLAB for numerical testing. The branch current phasors and the PMU measurements are obtained from MATPOWER [27]. To solve the centralized algorithm in Equation (7), we used CVX, a package for specifying and solving convex optimization problems [28]. The PMU measurement noise is simulated as an independent zero-mean Gaussian with its covariance matrix **Λ_n_** = 0.002**I_n_**. The covariance matrix **Λ_p_** is set to 0.003**I_p_**, where **I_p_** is an identity matrix with the same dimension as the unknown vector.

### 6.1. WSCC Nine-Bus Test Case

**Figure 3.**
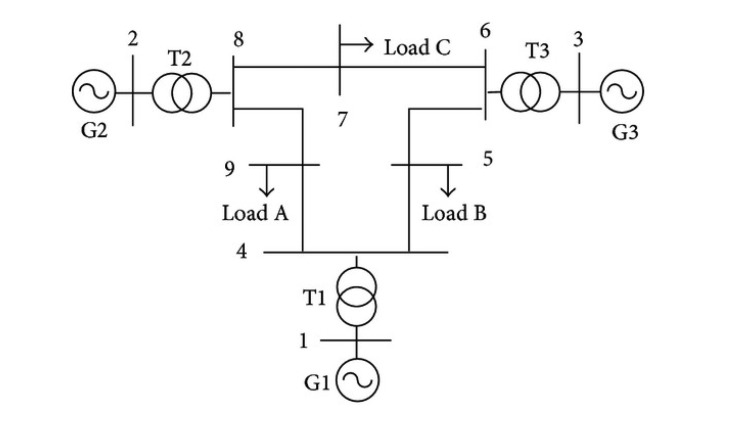
WSCC nine-bus test case system.

In this section, the WSCC nine-bus test case system was used for our simulation. The diagram of the system is demonstrated in Figure 3. There are three generators (G1,G2,G3), three transformers (T1,T2,T3) and nine lines in which the line parameter information is listed in Table 1.

**Figure 4.**
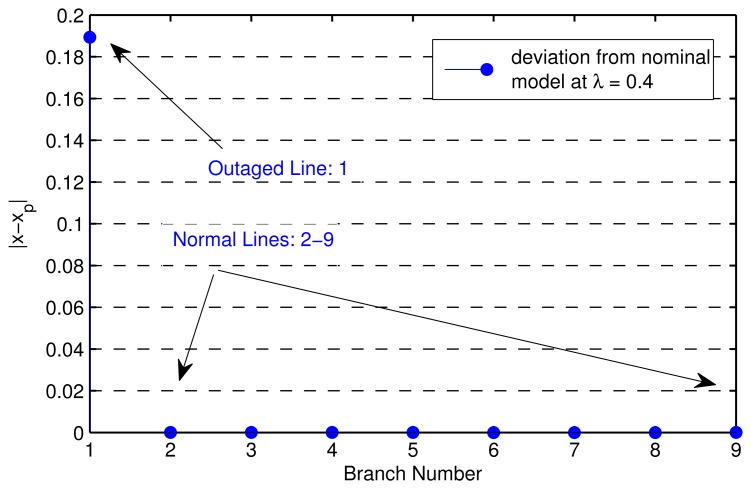
Centralized line outage detection.

From Table 1, the line-to-bus admittance matrix **Y_fl_** can be formed, which is used for constructing the measurement matrix **H** in Equation (7). In this case, the size of the unknown vector is nine by one, and we place two PMUs at Bus 4 and Bus 6 with their line current measurements in (1–4), (4–5), (9–4), (5–6), (3–6), (6–7). The system is assumed to be at steady state before and after the line change. We made the line change on the reactance of line (1–4), which was altered from 0.0576 to infinity. Then, we ran a DC power flow in MATPOWER to obtain the branch currents in normal conditions and the measurements after change. The above are all of the quantities considered as the input to our centralized line change detection algorithm. The result in Figure 4 shows that the faulty line (1–4) has been correctly detected by the algorithm. Note that here, λ from 0.35–0.45 can guarantee the accurate decision in this case.

**Figure 5.**
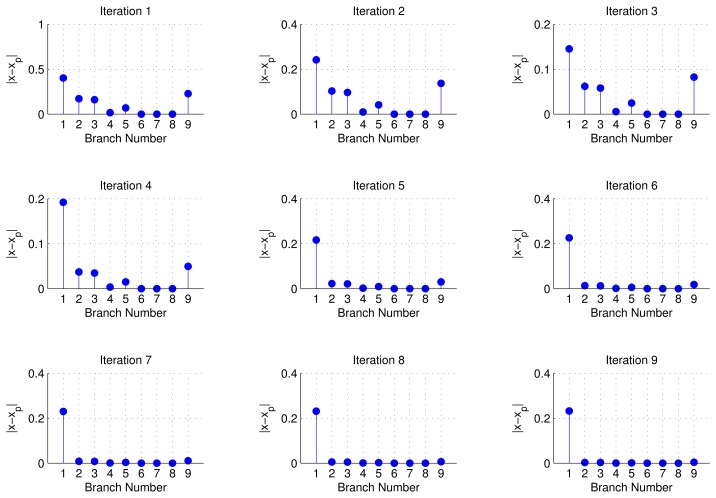
Distributed line outage detection.

We also tested our D-LCD algorithm on this nine-bus system, and the results of first nine ADMM iterations are captured in Figure 5. Note that initially, Branches 1–3, 5 and 9 have positive values, which means that they are all seen as a group of possible faulty lines. During Iteration 2–4, the values of Branches 1–3, 5 and 9 are actually decreasing, while an interesting point is that the decreasing speed of Branches 2, 3, 5 and 9 is much faster than Branch 1's. This observation is conformed with the theory part discussed previously, that the most likely set of branches should survive for the next iteration. From Iteration 5, Branch 1 is almost the only one standing out. This implies that Branch 1 is considered to be faulty by our distributed line change detection algorithm. In other words, the distributed algorithm almost converges to the centralized version result (we assume it as a benchmark) in Figure 4 in just five iterations.

### 6.2. IEEE 118-Bus System

**Figure 6.**
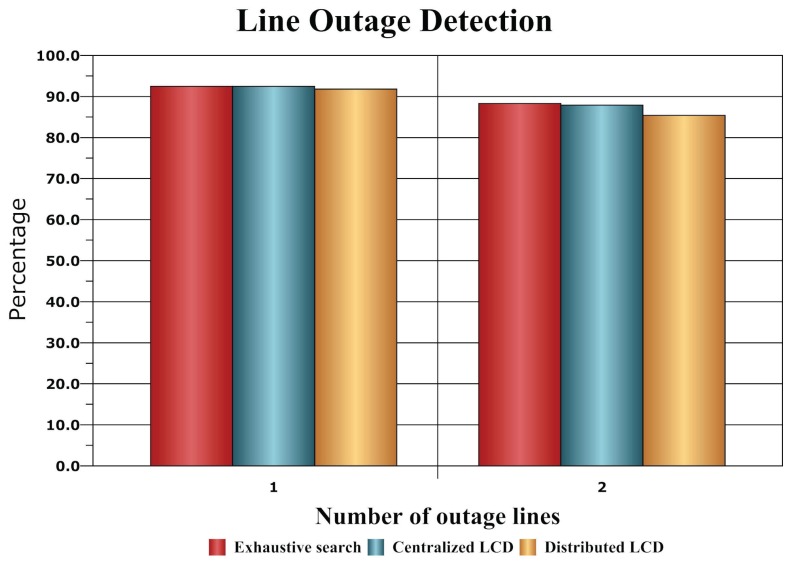
Comparison of detection performance for the IEEE 118-bus system.

The IEEE 118-bus system is tested here for evaluating our algorithms in the case of a large network. There are 186 branches in the test system, which will result in over 17, 200 possible faulty topologies in just a double-line outage scenario. All of the single line outage possibilities and 300 double-line outage cases are randomly chosen for testing. We adopt the method in [29] as our pool of measurements and randomly select two thirds the number of measurements from it. The exhaustive search algorithm in [6,7] is compared with our proposed methods in Figure 6 in terms of the percentage of the correctly detected outage pattern.

Note that the exhaustive search scheme is considered as the benchmark here since it is “optimal” in the statistical sense. It is impressive that both the centralized and distributed line outage detection methods perform very close to this optimal criterion.

### 6.3. IEEE 300-Bus System

The running times of the developed algorithms are also tested on the IEEE 300-bus test system. Following a Monte Carlo simulation method, the results for single and double-line outages are listed in Table 2.

In both the single- and double-line outage cases, D-LCD and D-LCD with warm start outperform the rest of algorithms, which is within expectations. It is found that as the system size and the number of line outages increases, the advantage of the warm-started D-LCD over distributed LCD becomes more sharper in terms of computational time. However, the exhaustive search approach does not scale well, as its running time jumps up in an order much higher than the others.

## 7. Discussion

In this paper, the proposed algorithms are assumed to work in transmission networks. Nevertheless, theoretically, they can also apply to distribution networks. The current distribution networks usually lack measurements and have a low level of monitoring capabilities. As smart grids develop, the proposed algorithms have the potential to work in distribution networks once the the infrastructure of “smart” sensor networks has been deployed.

Our proposed distributed algorithms involve the communication of neighboring PDCs. Each PDC only communicates with its neighbors by its estimates of the shared unknown variables. Hence, if the PDC is unable to collect the neighbors's information, it will keep its value of estimates unchanged. In this paper, the proposed distributed algorithms are more robust than the centralized ones in the following sense: for the centralized algorithms, if the sole processing center is attacked or fails, all of the information will be lost, and the system cannot obtain a solution for the outage detection function. However, for the proposed distributed algorithms, the probability of having similar serious conditions is much smaller.

## 8. Conclusions and Future Work

A novel distributed line outage detection algorithm was developed based on WAMS, which has been an important component of smart grids. The proposed approach allows multiple line outage identification using limited PMU measurements. The feature of low-complexity distributed processing in the proposed framework can enhance the efficiency, security and privacy level in smart grid monitoring. Numerical tests demonstrated the merits of the proposed schemes in coordinating the discovery of multiple line outages in a power grid.

Future research directions include the design and analysis of the control strategy (considering the HVDCand FACTdevices involved) after the localization of the faults and developing asynchronous (the present paper is under a synchronous setting), distributed line outage detection algorithms, which are highly required in the environments of distributed systems, such as future smart grids.

## Figures and Tables

**Table 1.** Line parameters of the WSCC nine-bus system.

**Line**	**Resistance (p.u)**	**Reactance (p.u)**
1–4	0.0000	0.0576
4–5	0.0170	0.0920
5–6	0.0390	0.1700
3–6	0.0000	0.0586
6–7	0.0119	0.1008
7–8	0.0085	0.0720
8–2	0.0000	0.0625
8–9	0.0320	0.1610
9–4	0.0100	0.0850

**Table 2.** Running time comparison for the IEEE 300-bus system.

**Algorithm**	**Single-Line Outage**	**Double-Line Outage**
Exhaustive Search	0.50 s	28 s
Centralized LCD	0.37 s	0.95 s
Distributed LCD	0.12 s	0.31 s
Warm-started D-LCD	0.053 s	0.14 s
